# Cell-Passage Activity Is Required for the Malarial Parasite to Cross the Liver Sinusoidal Cell Layer

**DOI:** 10.1371/journal.pbio.0020004

**Published:** 2004-01-20

**Authors:** Tomoko Ishino, Kazuhiko Yano, Yasuo Chinzei, Masao Yuda

**Affiliations:** **1**Mie University School of MedicineMieJapan; **2**Core Research for Evolutional Science and Technology (CREST), Japan Science and Technology Agency (JST)Kawaguchi, SaitamaJapan

## Abstract

Liver infection is an obligatory step in malarial transmission, but it remains unclear how the sporozoites gain access to the hepatocytes, which are separated from the circulatory system by the liver sinusoidal cell layer. We found that a novel microneme protein, named sporozoite microneme protein essential for cell traversal (SPECT), is produced by the liver-infective sporozoite of the rodent malaria parasite, Plasmodium berghei. Targeted disruption of the *spect* gene greatly reduced sporozoite infectivity to the liver. In vitro cell invasion assays revealed that these disruptants can infect hepatocytes normally but completely lack their cell passage ability. Their apparent liver infectivity was, however, restored by depletion of Kupffer cells, hepatic macrophages included in the sinusoidal cell layer. These results show that malarial sporozoites access hepatocytes through the liver sinusoidal cell layer by cell traversal motility mediated by SPECT and strongly suggest that Kupffer cells are main routes for this passage. Our findings may open the way for novel malaria transmission-blocking strategies that target molecules involved in sporozoite migration to the hepatocyte.

## Abstract 2

To infect hepatocytes, malarial sporozoites must traverse Kupffer cells of the liver. This active process is shown to be mediated by a newly identified Plasmodium protein called SPECT

## Introduction

Malaria is one of the most devastating infectious diseases in the world, killing more than 1 million people per year. Malaria is transmitted by bites of infected mosquitoes that inject sporozoites under the skin. The first obligatory step for these parasites to establish infection in humans is migration to hepatocytes, where they proliferate and develop into the erythrocyte-invasive form (Sinnis 1996). This liver-invasive stage has been demonstrated as a promising target for antimalarial strategies that aim to establish sterile immunity against the malarial parasite (Nussenzweig et al. 1967; Hoffman et al. 1996). However, the mechanisms underlying the parasite's liver infection are largely unknown. In particular, it has been controversial how sporozoites reach the hepatocytes that are separated from blood circulation by the liver sinusoidal layer. The routes the sporozoites use to cross this layer, the modes of motility on which their migration is based, and the molecules of the parasite involved in this process are poorly understood.

Malarial parasites develop into sporozoites in the mosquito midgut and then invade the salivary gland, where they wait to be transferred to the mammalian host (Menard 2000). Once injected by mosquito bites under the skin, sporozoites enter the blood circulation and are carried to the liver by the bloodstream (Sinnis and Nussenzweig 1996; Menard 2000; Mota and Rodriguez 2002). In the liver, they are thought to be arrested on the inner surface of the liver sinusoidal vein and then leave the vein and infect the hepatocytes by crossing the sinusoidal wall (Sinnis and Nussenzweig 1996). This wall is a single-cell layer mainly composed of sinusoidal endothelial cells and Kupffer cells, which are hepatic macrophages. Several models have been proposed to explain how sporozoites cross this layer. Some authors proposed that sporozoites infect hepatocytes after crossing the liver endothelial cell through fenestrations in this cell (Vanderberg and Stewart 1990), but these openings are too small for sporozoites to freely pass through (Mota and Rodriguez 2002). Other authors have suggested that Kupffer cells are gates for sporozoites to access hepatocytes, based on the ultrastructural observation that sporozoites were found inside Kupffer cells shortly after intravenous inoculation (Mota and Rodriguez 2002). This Kupffer cell hypothesis, however, has not been convincingly demonstrated, because other tools for probing into this event were lacking. Furthermore, the observation that the sporozoites in Kupffer cells sometimes have a vacuole around them makes the conclusion uncertain. Some authors have proposed that sporozoites are passively engulfed by Kupffer cells and then carried to the hepatocyte (Meis et al. 1983), and some have proposed that this migration is based on active motility accompanied by vacuole formation (Pradel and Frevert 2001).

The malarial parasite has no locomotory organelles such as flagella or cilia. Motility of the host-invasive stages of the malarial parasite, including the sporozoite, is dependent on secretion of micronemes that are organelles occupying the cytoplasm of the parasite (Sultan 1999; Menard 2001). Micronemal components, which may include several attachment proteins, are secreted from the apical pore during parasite movement and are translocated backwards along the parasite cell surface by actomyosin motors of the parasite. This surface movement is believed to generate traction for parasite-invasive motility.

Salivary gland sporozoites display three modes of motility in vitro dependent on secretion of micronemes (Mota and Rodriguez 2002). One mode is gliding motility on a solid surface, which can be observed as circular movement on a glass slide, probably representing gliding motility on the cell surface. The other two are cell-invasive motilities: cell-infection and cell-traversal motility (Mota et al. 2001; Kappe et al. 2003). Cell-infection motility is accompanied by vacuole formation and is followed by parasite development into exoerythrocytic forms (EEFs). Cell-traversal motility, on the other hand, involves plasma-membrane disruption and is followed by migration through the cytoplasm and eventual escape from the cell. Recently, Mota et al. (2002) revealed that this type of cell-invasion motility can be identified by conventional cell-wound assay. According to the observation that passage through some hepatocytes by this motility precedes hepatocyte infection, they proposed the hypothesis that this motility is necessary for sporozoites to be activated for hepatocyte infection (Mota et al. 2002). However, the role of this motility in liver infection remains unclear.

Aiming at identification of molecules involved in sporozoite infection, we screened an expressed sequence tag (EST) database of the salivary gland sporozoite of a rodent malarial parasite, Plasmodium berghei. In this paper, we report a novel microneme protein, named SPECT (sporozoite microneme protein essential for cell traversal), which is specifically produced by the liver-infective sporozoite and is essential for the sporozoite's cell-passage ability. By using *spect*-disrupted parasites, we show that cell-passage ability of the sporozoite plays a critical role in malarial transmission to the vertebrate host and is required for sporozoites to access hepatocytes by traversal of the liver sinusoidal cell layer. In addition, we provide a model of sporozoite liver infection, which suggests an answer to the question of how sporozoites reach the hepatocytes.

## Results

### Identification of cDNA Encoding SPECT from P. berghei Salivary Gland Sporozoite EST Database

**Figure 1:**
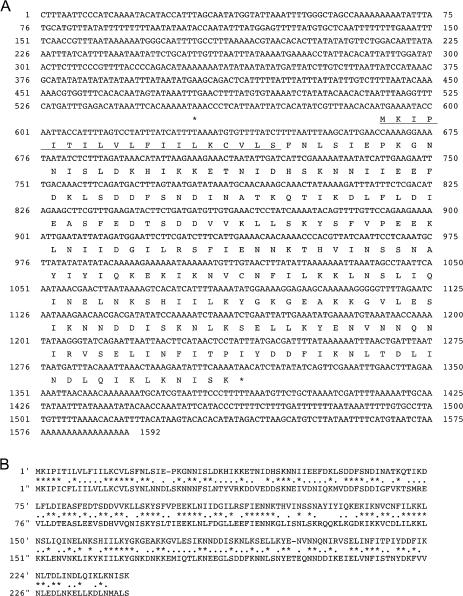
Sequence Analysis of *spect* cDNA (A) Nucleotide sequence of *spect* cDNA (top lane) and the deduced amino acid sequence (bottom lane) are shown. The predicted N-terminal signal sequence is underlined. The numbers indicate positions of the nucleotides starting from the 5′ end. The asterisks indicate the termination codon. (B) A comparison of deduced amino acid sequences of *P. berghei spect* (top) and *P. falciparum spect* (bottom). Gaps are introduced to obtain optical matching by using GENETIX-MAC software. Asterisks or dots show conserved or similar residues, respectively. The amino acid numbers from the first Met residue are shown on the left of each line.

Sporozoites acquire the ability to infect the mammalian liver after infection of the mosquito salivary glands (Sultan et al. 1997), indicating that novel protein synthesis for liver infection begins in this stage (Matuschewski et al. 2002). To search for malarial genes involved in liver infection, we screened an EST database of P. berghei salivary gland sporozoites. We assembled 3,825 ESTs, obtained 502 contigs, and screened them for genes encoding secretory proteins or membrane-associated proteins, which may participate in host–parasite interactions. This screening was started from the contigs containing a high number of ESTs, since the number of ESTs may correlate with the expression levels of the respective genes. In this process, we identified a contig composed of ten ESTs, encoding a putative secretory protein of 241 amino acids (Figure 1A). The expected molecular mass for the N-terminal signal sequence-processed form of this protein was 25 kDa. We named this protein SPECT (sporozoite microneme protein essential for cell traversal), since it is essential for sporozoite passage through a host cell, as described later.

Southern blot analysis showed that the *spect* gene is a single-copy gene (data not shown). Sequence analysis of the *spect* gene identified four introns (data not shown). A computer search of *Plasmodium* genome databases (Carlton et al. 2002; Gardner et al. 2002) revealed that this gene is conserved through several *Plasmodium* species. The orthologous protein in *P. falciparum,* the clinically most important human malaria parasite, shared 45.6% sequence identity with P. berghei SPECT (Figure 1B).

### SPECT Is Produced Specifically by Salivary Gland Sporozoites and Localized in Micronemes

**Figure 2:**
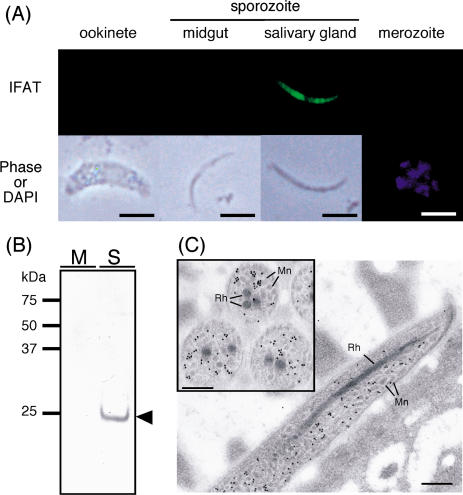
SPECT Is a Microneme Protein Specifically Produced in the Liver-Infective Sporozoite Stage (A) Indirect immunofluorescence microscopy of all four invasive forms of the malarial parasite (indicated over the panel). Parasites were stained with primary antibodies against SPECT, followed by FITC-conjugated secondary antibodies. SPECT was detected only in the salivary gland sporozoite, the liver-infective stage. The corresponding phase-contrast or DAPI-stained image (Phase or DAPI) is shown under each image. Scale bars, 5 μm (B) Western blot analysis of SPECT production in the midgut sporozoite (M) and the salivary gland sporozoite (S). Lysate of 500,000 sporozoites was loaded onto each lane and detected with the same antibody used in (A). SPECT was detected as a single band of 22 kDa (arrowhead) only in the salivary gland sporozoite. (C) Immunoelectron microscopy of sporozoites in the salivary gland. Ultrathin sections of a mosquito salivary gland infected with sporozoites were incubated with the same antibody used in (A) followed by secondary antibodies conjugated with gold particles (15 nm). Particles were localized to micronemes (Mn) but not to rhoptories (Rh). Axial (inset) and vertical images are shown. Scale bars, 0.5 μm.

The expression profile of this gene in the malarial life cycle was investigated. Immunofluorescent analysis in all host-invasive stages showed that SPECT production was restricted to sporozoites in the salivary gland (Figure 2A). It is noteworthy that SPECT is not detected in sporozoites in the midgut, because this expression profile strongly suggests that SPECT is specifically involved in liver infection. Western blot analysis revealed SPECT as a 22 kDa protein in salivary gland sporozoites, but not in midgut sporozoites (Figure 2B), confirming that SPECT is produced after invasion into the salivary gland. Immunoelectron microscopy showed that SPECT is localized in the sporozoite to micronemes that are secretory organelles occupying the cytoplasm (Figure 2C). Micronemes are common to motile stages of *Plasmodium* parasites and play a central role in host-invasive motility (Sultan 1999; Menard 2001). Taken together, these results indicate that SPECT plays a role in the liver-invasive motility of the sporozoite.

### SPECT Plays an Important Role in Sporozoite Infection of the Host Liver

**Figure 3:**
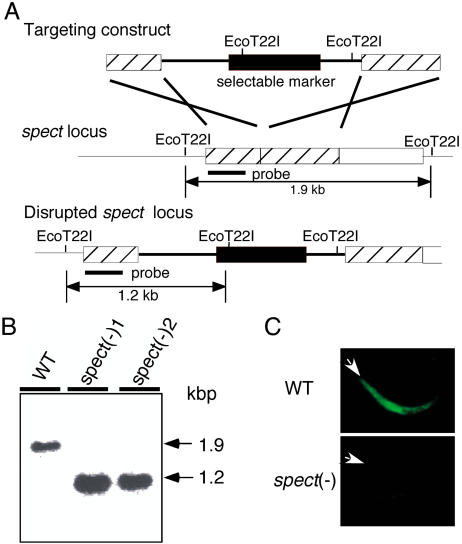
Targeted Disruption of the *spect* Gene (A) Schematic representation of targeted disruption of the *spect* gene. The targeting vector (top) containing a selectable marker gene is integrated into the *spect* gene locus (middle) by double crossover. This recombination event resulted in the disruption of the *spect* gene and confers pyrimethamine resistance to disruptants (bottom). (B) Genomic Southern blot hybridization of wild-type (WT) and *spect*(−) populations. Genomic DNA isolated from the respective parasite populations was digested with EcoT22I and hybridized with the probe indicated in (A) by a solid bar. By integration of the targeting construct, the size of detected fragments was decreased from 1.9 kbp to 1.2 kbp. The result is shown for two independently prepared populations, *spect*(−)1 and *spect*(−)2. (C) Immunofluorescence microscopy of the wild-type (WT) and *spect*(−) parasite. Sporozoites were collected from the salivary gland and stained with primary antibody against SPECT followed by FITC-conjugated secondary antibodies. The apical end of each sporozoite is indicated by an arrowhead.

To investigate the function of SPECT protein, we generated *spect*-disrupted parasites by homologous recombination (Figure 3A). The *spect* disruptants were selected by the antimalarial drug pyrimethamine and were separated from wild-type parasites by limiting dilution. Disruption of the *spect* locus was confirmed by Southern blot analysis (Figure 3B). To exclude the possibility that the *spect*-disrupted populations obtained were derived from a single clone, two independently obtained *spect*-disrupted populations (*spect*(−)1 and *spect*(−)2) were used in the following experiments.

In the intra-erythrocytic stage, SPECT gene disruption did not affect parasite proliferation, as the growth rates in rat blood were almost the same in the *spect*-disrupted and wild-type populations (data not shown). Furthermore, disruption of the gene did not affect parasite development in the mosquito vector, as numbers of sporozoites residing in the midgut and in the salivary glands were similar in the *spect*-disrupted and wild-type populations (Table 1).

**Table 1:**
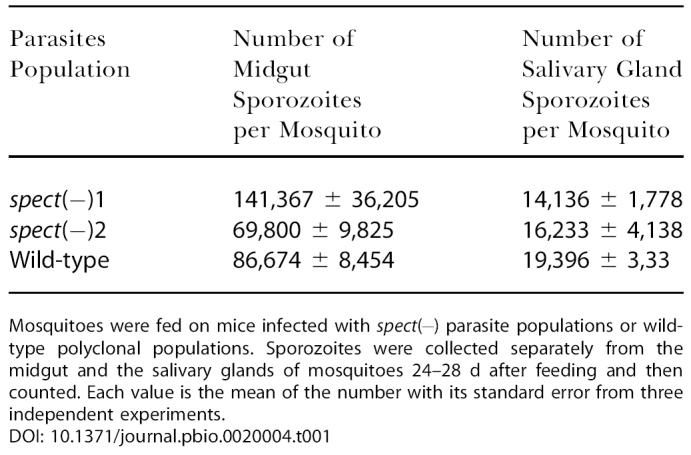
SPECT Disrupted Parasites Develop Normally into Sporozoites and Invade the Salivary Gland in the Mosquito Vector

Mosquitoes were fed on mice infected with spect(−) parasite populations or wild-type polyclonal populations. Sporozoites were collected separately from the midgut and the salivary glands of mosquitoes 24–28 d after feeding and then counted. Each value is the mean of the number with its standard error from three independent experiments

**Figure 4:**
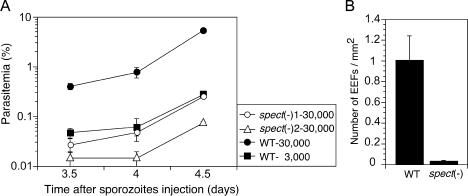
Targeted Disruption of *spect* Results in Reduction of Sporozoite Infectivity to the Liver (A) The salivary gland sporozoites of each parasite population were injected intravenously into five rats. The parasitemia of each rat was checked by a Giemsa-stained blood smear after inoculation on the days indicated. The average parasitemia after inoculation of 30,000 sporozoites was significantly low in disruptant populations, whereas their growth rates in the blood were essentially the same as the wild-type. The numbers of parasites inoculated were as follows: 30,000 *spect*(−)1 (open circles), 30,000 *spect*(−)2 (open triangles), 30,000 wild-type (filled circles), and 3,000 wild-type (filled squares). Values shown represent the mean parasitemia (± SEM) of five rats. (B) The salivary gland sporozoites (500,000) of wild-type or *spect*-disrupted parasites were inoculated intravenously into 3-wk-old rats. After 24 h, the livers were fixed with paraformaldehyde and frozen. The number of EEFs on each cryostat sections was estimated by indirect immunofluorescence analysis using anti-CS antiserum. Values shown represent the mean number of EEFs per square millimeter (± SEM) of at least three rats.

Next, the liver infectivity of the *spect*-disrupted sporozoites was examined. Rats were intravenously inoculated with sporozoites, and the progress of parasitemia, the percentage of infected erythrocytes, was measured in the exponential growth period (from 3.5 d to 5 d after inoculation). It is thought that the parasitemias reflect the liver infectivity of the respective parasite populations, since the growth rates of their intraerythrocytic stages are similar (shown by the parallel slopes of the increase in parasitemia in Figure 4). Based on the average parasitemia at 3.5 d after inoculation of 30,000 sporozoites, the liver infectivities of the two disruptant strains were estimated to be 15- and 28-fold lower, respectively, than that of the wild-type. These results are consistent with the observation that the parasitemias after injection of 30,000 disruptant sporozoites were lower than that from 3,000 wild-type sporozoites.

The liver infectivity was also evaluated by the number of early EEFs. Frozen sections of the rat liver was prepared 24 h after sporozoite injection and EEFs were counted by immunofluorescence microscopy. As shown in Figure 4B, EEFs were approximately 30-fold decreased by *spect* gene disruption. This reduction rate agrees well with that estimated by parasitemia. These results indicate that SPECT plays a role in the process of sporozoite invasion into the liver.

### SPECT Is Essential for Sporozoite Cell-Passage Ability

**Figure 5:**
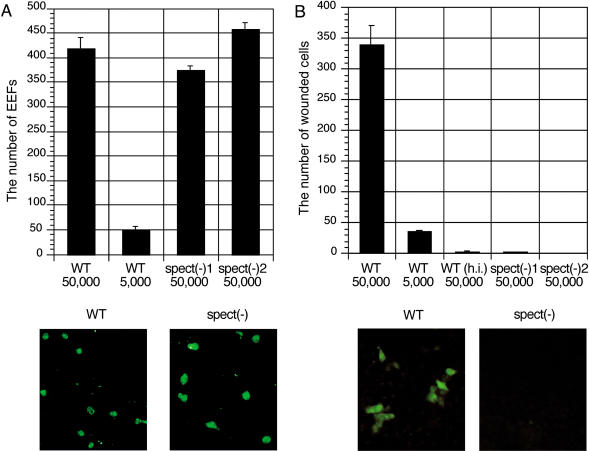
*spect* Disruption Results in Loss of Cell-Passage Activity of the Sporozoite (A) *spect* disruption does not affect sporozoite ability to infect hepatocytes. (Top panel) Comparison of EEF numbers between disruptants (*spect*(−)) and wild-type (WT) parasites. Salivary gland sporozoites were added to HepG2 cells and cultured for 48 h. EEFs formed were counted after immunofluorescence staining with an antiserum against CS protein. (Bottom panels) Representative fluorescence stained images. (B) Sporozoites lacking SPECT cannot traverse HeLa cells. (Top) Comparison of cell-passage activity between disruptants and wild-type parasites. Salivary gland sporozoites were added to HeLa cells and incubated for 1 h with FITC-conjugated dextran (1 mg/ml). Cell-passage activity was estimated by the number of cells wounded by sporozoite passage, which were identified by cytosolic labeling with FITC-conjugated dextran. (Bottom) Representative fluorescence stained images. All data are mean numbers of EEFs or FITC-positive cells in a one-fifth area of an 8-well chamber slide with standard errors for at least three independent experiments.

Localization of SPECT in micronemes indicates its involvement in the invasive motility of the sporozoite. The motility of *spect*-disrupted sporozoites was investigated by three in vitro assays corresponding to three modes of motility of the sporozoite. First, we checked gliding motility on a solid surface, which is essential for sporozoite infectivity. Most disruptants displayed a typical circular movement, and the proportion of motile sporozoites was almost identical in disruptant and wild-type parasites (63.6% and 67.5%, respectively), showing that their gliding motility is not affected by SPECT gene disruption. Second, we examined the ability of the sporozoites to infect hepatocytes. This was assayed by formation of EEFs in a human hepatoma cell line, HepG2 (Hollingdale et al. 1981). As shown in Figure 5A, the disruptants formed EEFs in similar numbers to the wild-type, indicating that they retain normal infectivity to the hepatocyte. Third, we examined cell-traversal ability that takes place prior to hepatocyte infection. This was estimated by the number of membrane-wounded cultured cells that were labeled by uptake of fluorescein isothiocyanate (FITC)-conjugated dextran from the medium (Mota et al. 2001). As shown in Figure 5B, the cell-wound assay using HeLa cells showed that the disruptants lost their cell-passage activity completely. The same results were obtained in HepG2 cells (data not shown). These results revealed that SPECT is specifically involved in cell-traversal ability and suggest that lack of this ability reduced liver infectivity of the disruptants.

### Cell Passage Ability Is Necessary for Sporozoites to Traverse the Sinusoidal Layer Cells and to Access Hepatocytes

**Figure 6:**
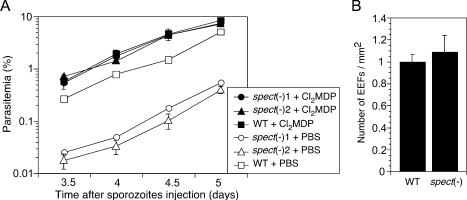
Restoration of *spect*(−) Sporozoite Infectivity in Kupffer Cell-Depleted Rats (A) Liposome-encapsulated Cl_2_MDP (filled points) or PBS (open) was injected intravenously into rats. After 48 h, 30,000 sporozoites of *spect*(−)1 (circles), *spect*(−)2 (triangles), or wild-type (squares) populations were inoculated intravenously. Parasitemia of each rat was checked by Giemsa-stained blood smears after inoculation on the days indicated. Values shown represent the mean parasitemia (± SEM) of five rats. (B) Salivary gland sporozoites (500,000) of each parasite population were inoculated intravenously into Kupffer cell-depleted rats. After 24 h, the livers were fixed with paraformaldehyde and frozen. The number of EEFs on each cryostat section was estimated by indirect immunofluorescence analysis using anti-CS antiserum. Values shown represent the mean number of EEFs per square millimeter (± SEM) of at least three rats.

To access the hepatocytes, sporozoites must cross the sinusoidal layer, which separates them from the circulation. We assumed that SPECT was necessary for this process. Since Kupffer cells are major components of this layer and have been reported as the main gates for sporozoite access to the hepatocyte, we prepared Kupffer cell-depleted rats by intravenous injection of liposome-encapsulated dichloromethylene diphosphonate (Cl_2_MDP) (Vreden et al. 1993; van Rooijen and Sanders 1994) and tested them for infection by disruptant and wild-type sporozoites. As shown in Figure 6A, infectivities of *spect*-disruptants assessed by parasitemia were increased by 22- and 53-fold by Kupffer cell depletion and, as a result, became equal to that of the wild-type. The numbers of early EEFs detected in the liver sections were also almost identical in wild-type and *spect*-disrupted parasites (Figure 6B). These results show that the disruptants' loss of infectivity is localized at the sinusoidal cell layer and that the cell-passage ability of the sporozoite is necessary to cross this layer and, specifically, the Kupffer cells.

## Discussion

It has been reported that the *Plasmodium* sporozoite has the ability to traverse cultured cells rapidly (Mota et al. 2001), but the role of this process in liver infection has remained unclear. On the other hand, it is poorly understood how the sporozoite migrates from the circulatory system to the hepatocyte. In this paper, we address these issues using a gene-targeting technique. We have shown that the cell-traversal activity of the sporozoite is necessary for it to leave the circulatory system by crossing the liver sinusoidal cell layer. These results are the first to reveal the role of cell-traversal activity in malarial transmission.

In vitro cell invasion assays showed that *spect*-disrupted sporozoites completely lose cell passage activity, but preserve normal infectivity to the hepatocyte (see Figure 5). These results clearly demonstrated that these two cell-invasion activities are independent of each other. This conclusion contradicts the hypothesis proposed by Mota et al. (2002) that cell passage activates the sporozoite for hepatocyte infection. They assumed that sporozoites traverse some hepatocytes before infecting a hepatocyte and that this passage alters their mode of cell invasion from passage to infection (Mota et al. 2002). Our results, however, demonstrated that lack of previous cell passage has no influence on the infectivity to hepatocytes. This independence was confirmed in vivo by the result that disruptants and wild-type showed the same liver infectivities in Kupffer cell-depleted rats (see Figure 6). Therefore, sporozoites may change their mode of invasive motility according to other factors, which remain to be elucidated. We suppose that secretion of the micronemal contents during gliding on the cell surface might be one such factor, since this motility may precede hepatocyte infection as discussed below.

Our results indicate that the liver sinusoidal barrier is not perfect, since a small proportion of the *spect*-disrupted sporozoites can infect the liver (see Figure 4). It is supposed that this layer may have a few openings and the disruptants can migrate through them by gliding along the epithelial cell surface. In Kupffer cell-depleted rats, on the other hand, both disruptants and wild-type may migrate through the numerous gaps created among the endothelial cells, resulting in elimination of the phenotypic difference. Since Kupffer cells constitute approximately 30% of the sinusoidal cells (Bouwens et al. 1986), their depletion from this layer may leave many gaps that cannot readily be repaired. Supposedly, sporozoites cross these gaps in the same way as they migrate through the few gaps in normal rats.

Experiments using Kupffer cell-depleted rats indicate that Kupffer cells are not involved in sporozoites targeting the liver, because the depletion did not reduce the susceptibility of rats to sporozoite infection. Thus, sporozoites seem to be first arrested on the endothelial cell surface or on the glycosaminoglycans extending through endothelial fenestrations and then migrate to Kupffer cells (Cerami et al. 1992; Pradel et al. 2002). If so, gliding motility on the cell surface would be necessary for the sporozoite to migrate from initial attachment sites to Kupffer cells (or to gaps) along the inner surface of the sinusoidal layer as well as for the sporozoite to migrate through gaps. These assumptions imply that after Kupffer cell depletion, sporozoites can arrive at the hepatocyte by gliding motility alone, in accord with the observation that the disruptants can infect Kupffer cell-depleted rats with the same infectivity as the wild-type.

**Figure 7:**
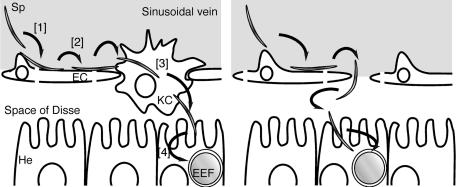
Schematic Representation of Sporozoite Migration to and Infection of Hepatocytes (Left) Sporozoites migrate to the space of Disse through the Kupffer cells. [1] The sporozoite (Sp) in the circulatory system is sequestered to the sinusoidal endothelial cell (EC) by specific recognition of the cell surface or glycosaminoglycans extending from the hepatocytes (He) through fenestration. [2] The sporozoite begins to glide on the epithelial cell surface. [3] Encountering a Kupffer cell (KC), the sporozoite ruptures the plasma membrane, passes through the cell, and enters into the space of Disse. Thus, the sporozoite gains access to hepatocytes. This step requires SPECT. [4] The sporozoite infects a hepatocyte with formation of a vacuole and develops into EEF in the hepatocyte. (Right) An alternative route to the hepatocyte. A small number of sporozoites, which find gaps in the sinusoidal layer while gliding, migrate to hepatocytes directly through the openings without need for cell passage and infect the hepatocytes. Likewise, in Kupffer cell-depleted rats, both wild-type and *spect*(−) sporozoites can enter hepatocytes through numerous gaps present between the sinusoidal endothelial cells.

Our results strongly suggest that Kupffer cells are main gates for sporozoites to access hepatocytes. Previous electron microscopic studies have reported that sporozoites are observed in Kupffer cells after intravenous inoculation, and some of them are found within vacuoles (Meis et al. 1983; Pradel and Frevert 2001). Based on this observation, it has been speculated that sporozoites invade the Kupffer cell by a motility distinct from passage that does not involve parasitophorous vacuole formation. Our results, on the contrary, indicate that sporozoites cross the layer by the same cell-passage motility as observed in vitro. We think this discrepancy indicates the following two possibilities. One is that the vacuole formed in the Kupffer cell after rupture of its cell membrane is different from the parasitophorous vacuole formed in the hepatocyte, although their differences cannot be distinguished by electron microscopy. Another possibility is that the parasites seen in vacuoles were phagocytosed ones and not in the process of invasion. In fact, if their invasion mode is cell-traversal motility, as we believe, this event may be rapidly completed and difficult to catch by electron microscopy. Therefore, many phagocytosed parasites could be included among those seen. Taking the evidence together, we propose that the sporozoites access the hepatocyte through Kupffer cells by the same cell-traversal motility that has been identified in vitro, and we propose a model for sporozoite liver infection in Figure 7.

In this study we have established the significance of cell-passage ability of the sporozoite in malaria transmission and have demonstrated that this ability is necessary for breaking through the liver sinusoidal barrier. Cell-traversal activity plays an important role in other invasive stages of the malarial parasite, including the ookinete, which migrates through the epithelial cells of the mosquito midgut, and the sporozoite in the oocyst, which is released from the mature oocyst and then migrates through the salivary gland cell. Our study revealed that another cellular barrier is present in the malarial life cycle and sporozoites must break through this barrier by cell-traversal activity.

Our recent work has identified two other genes that are involved in the cell passage activity of the sporozoite. Like SPECT, the products of these genes have a secretory protein-like structure and are localized in the micronemes. Furthermore, sporozoites disrupted for these genes have similar phenotypic character to *spect*-disrupted ones, including impaired cell-passage ability, decreased liver infectivity with similar reduction rate, complete restoration of the infectivity in Kupffer cell-depleted rats, normal gliding motility, and normal hepatocyte infectivity (unpublished data). This suggests that the cell-traversal ability of the sporozoite is realized by cooperation of several microneme proteins. We suggest that these molecules could be targets for antimalarial strategies, since success in crossing this layer is critical for the malarial parasite to establish infection in humans. Elucidation of the molecular mechanisms of passage may lead to novel malaria transmission-blocking strategies that prevent sporozoites from gaining access to the hepatocyte.

## Materials and Methods

### 

#### Parasite preparations

Female 6–10-wk-old BALB/c mice (Japan SLC, Inc., Hamamatsu, Japan) infected with the P. berghei ANKA strain were prepared by peritoneal injection of infected blood that was stored at −70°C. For the purification of sporozoites, infected mosquitoes were dissected 24–28 d after the infective blood meal. The salivary glands and midgut were separately collected in medium 199 on ice and then gently ground to release the sporozoites. Ookinetes and erythrocytic-stage parasites were prepared as described previously (Yuda et al. 1999; Kariu et al. 2002).

#### Genomic Southern blot hybridization

Genomic DNA of P. berghei parasites (2 μg) was digested with ClaI, EcoRI, EcoT22I, HindIII, or XbaI, separated on 1.2% agarose gel and then transferred to a nylon membrane. DNA fragments were amplified by PCR using genomic DNA as template with the following primers: 5′-TGGGCAATTTTGCCTTTAAAAACG-3′ and 5′-TTTCATTGTGTTAAACGATAAGTG-3′. They were labeled with [^32^P]dCTP and used as probes.

#### Antibody preparation and Western blot analysis

Recombinant SPECT without signal sequence was expressed as a glutathione S-transferase (GST)–fusion protein using the pGEX 6p-1 system (Amersham Bioscience, Uppsala, Sweden). The recombinant protein was purified with a GST column and used for immunization of rabbits. Specific antibodies were affinity purified using a N-hydroxysuccinimide-activated column (Amersham Bioscience) coupled with recombinant SPECT protein. For CS antiserum production, the peptide DPPPPNANDPAPPNAN, corresponding to the repeat region, was conjugated to keyhole limpet hemocyanin as a carrier and used for the immunization of rabbits. Western blot analysis was performed as described previously (Kariu et al. 2002).

#### Immunofluorescence microscopy and immunoelectron microscopy

Immunofluorescence microscopy was performed as described previously (Kariu et al. 2002). Purified parasites were fixed in acetone for 2 min. The slides were incubated with anti-SPECT rabbit antibodies and then with FITC-conjugated secondary antibody (Zymed. South San Francisco, California, United States). For nuclear staining, 4′,6-diamidino-2-phenylindole (DAPI) (0.02 μg/ml final concentration) was added to the secondary antibody solution. Immunoelectron microscopy was performed as described previously (Yuda et al. 2001). In brief, purified parasites were fixed in 1% paraformaldehyde–0.1% glutaraldehyde for 15 min on ice. After embedding in LR Gold resin (London Resin Company Ltd., London, United Kingdom), ultrathin sections were incubated with anti-SPECT antibodies and then with secondary antibody conjugated to gold particles (15 nm diameter) (AuroProbe, Amersham Pharmacia Biotech, Uppsala, Sweden). The samples were examined with a Hitachi H-800 transmission electron microscope (Hitachi, Tokyo, Japan) at an acceleration voltage of 100 kV.

#### Targeted disruption of the *spect* gene

For construction of the targeting vector, two fragments of the *spect* gene were amplified by PCR using genomic DNA as template with the primer pairs 5′-CGCGAGCTCGCAATATGGTATTAAATTTTGGGCTAGCCA-3′ and 5′-CGCGGATCCGGTATTTTCATTGTGTTAAACGATATGTGA-3′ and 5′-CCGCTCGAGGTCCTATTTATCATTTTAAAATGTGTTTTATC-3′ and 5′-CGGGGTACCAATCGTCATAAATAGGAGTTATGAAGT-3′. These fragments were cloned into either side of the selectable marker gene in pBluescript (Strategene, La Jolla, California, United States). The gene targeting experiment was performed as described previously (Yuda et al. 1999).

#### Evaluation of sporozoite infectivity to rats

Sporozoites collected from mosquito salivary glands were suspended in medium 199 and then injected intravenously into 3-wk-old female Wistar rats (Japan SLC, Inc., Hamamatsu, Japan) (*n* = 5). Before each inoculation, sporozoites were checked for their ability to glide in vitro to confirm that they contained over 60% motile sporozoites. Parasitemia was checked every 12 h by a Giemsa-stained blood smear.

#### Measurement of the number of EEFs in the infected liver

Sporozoites (5.0 × 10^5^) were intravenously inoculated into a 3-wk-old female Wistar rat. After 24 h, the liver was perfused with PBS followed by 4% paraformaldehyde. The liver was further fixed in 4% paraformaldehyde for 6 h and frozen in liquid nitrogen. Cryostat sections (20 μm) were prepared from the left lobe and fixed in acetone for 2 min on a glass slide. The EEFs were detected by immunofluorescence staining using rabbit anti-CS antiserum and FITC-conjugated secondary antibody. At least 12 sections were examined under an Olympus (Tokyo, Japan) BX60 fluorescence microscope (200×) and the number of EEFs per square millimeter was calculated.

#### EEF development assay in vitro

The EEF formation assay was performed as described previously (Hollingdale et al. 1981) with minor modifications. HepG2 cells (5.0 × 10^5^) were plated in 8-well chamber slides. Sporozoites (5.0 × 10^3^ or 5.0 × 10^4^) were suspended in 100 μl of complete medium and added to this culture. After 2 h, the media were replaced with 400 μl of fresh complete medium supplemented with 3 μg/ml glucose. The slides were incubated for 2 d with medium changed twice a day and were fixed in acetone for 2 min. The EEFs were detected by immunofluorescence staining as described above. The number of EEFs in one-fifth of the area of each well was counted under an Olympus BX60 fluorescence microscope (200×).

#### Cell-traversing activity assay

The traversing activity of the sporozoite was examined using a standard cell-wounding and membrane repair assay (Mota et al. 2001). HepG2 cells (2.5 × 10^5^) or HeLa cells (5.0 × 10^4^) were inoculated into 8-well chamber slides (Nunc Inc., Napierville, Illinois, United States). Sporozoites were added 2 d later to cells for 1 h in the presence of 1 mg/ml FITC-labeled dextran (10,000 MW, lysine-fixable; Molecular Probes, Inc., Eugene, Oregon, United States). The cells were incubated for an additional 3 h in complete culture medium and fixed with 4% paraformaldehyde in PBS. The number of FITC-positive cells was counted under a fluorescence microscope.

#### Depletion of rat Kupffer cells

For depletion of Kupffer cells, 3-wk-old female Wistar rats were injected intravenously with 120 μl of liposome-encapsulated Cl_2_MDP or an equal volume of PBS as control. After 48 h, sporozoites were injected into a tail vein and the parasitemia was checked by Giemsa-stained blood smears. Cl_2_MDP liposomes were prepared as described elsewhere (van Rooijen and Sanders 1994). Elimination of Kupffer cells was confirmed by immunoperoxidase staining after liver perfusion with PBS followed by fixation with 4% paraformaldehyde in PBS. Cl_2_MDP was a gift from Roche Diagnostics (Mannheim, Germany).

## Abbreviations

Cl_2_MDP: dichloromethylene diphosphonate
CS: circum sporozoite protein
DAPI: 4′,6-diamidino-2-phenylindole
EEF: exoerythrocytic form
EST: expressed sequence tag
FITC: fluorescein isothiocyanate
GST: glutathione S-transferase
SPECT: sporozoite microneme protein essential for cell traversal
